# In the era of copy number variation sequencing: changes in the target population for prenatal diagnosis and what is the optimal prenatal diagnostic strategy?

**DOI:** 10.3389/fmed.2025.1610477

**Published:** 2025-12-17

**Authors:** Shaozhe Yang, Rongxiang Li, Shuwen Xin, Yanqi He, Shaoxia Teng, Yanan Gao, Xiuhong Fu

**Affiliations:** 1 Henan Key Laboratory of Fertility Protection and Aristogenesis, Luohe Central Hospital, Luohe, China; 2 Luohe Reproductive Medicine and Genetics Center, Luohe Central Hospital, Luohe, China

**Keywords:** copy number variant, copy number variant sequencing, karyotyping, prenatal diagnosis, prenatal diagnosis strategy

## Abstract

**Purpose:**

Copy number variation sequencing (CNV-Seq) has become a first-line prenatal diagnostic technology. The purpose of this study was to investigate the changes in the target population for prenatal diagnosis in the CNV-Seq era and to assess the clinical value and economic costs of combining the use of CNV-Seq with karyotyping.

**Methods:**

The prenatal diagnostic indications of 3,931 pregnant women (3,952 samples of amniotic fluid) in three groups were statistically analyzed. The detection rates (DRs) of karyotype or CNV-Seq for each indication were determined. The abnormal results from karyotyping or CNV-Seq were analyzed. The detection efficiencies of karyotyping and CNV-Seq for each type of chromosomal abnormality were compared. The DRs of chromosomal abnormalities and the economic costs of the three prenatal diagnosis strategies were assessed.

**Results:**

Ultrasonic anomalies were the leading indications for the occurrence of copy number variation in prenatal diagnosis. The DRs for chromosomal abnormalities were 3.72% with karyotyping and 16.20% with CNV-Seq. The DRs of chromosomal abnormalities increased from 7.02% to 15.81% when CNV-Seq was combined with karyotyping in the Karyotype and CNV-Seq group. CNV-Seq confirmed the small supernumerary marker chromosome and unidentified karyotypes detected by karyotyping. Both karyotyping and CNV-Seq are capable of detecting low-level mosaicism.

**Conclusion:**

In the era of CNV-Seq, the target population for prenatal diagnosis is expanding. Combining CNV-Seq with karyotyping can increase the DRs of fetal chromosomal abnormalities. Therefore, combined testing is an efficient and relatively cost-effective prenatal diagnostic strategy.

## 1 Introduction

Birth defects are the leading factor behind early miscarriage, infant mortality, stillbirth, and congenital disabilities (1). The rate of birth defects in China is approximately 5.6%, with approximately 900,000 new cases reported each year, accounting for 20% of global birth defects (2). The serious impact of birth defects on the survival and quality of life of affected children results in immense suffering and economic burdens on both the children and their families. Chromosome abnormalities are responsible for more than 80% of genetic birth defects, specifically chromosomal numerical abnormalities, large fragment deletions/duplications, and genome copy number variations (CNVs) (3). Karyotyping of fetuses has long been considered the “gold standard” for diagnosing chromosomal abnormalities in fetuses (4). Karyotyping is used to detect most chromosomal abnormalities, including numerical abnormalities, large fragment deletions/duplications, and balanced structural rearrangements (such as balanced translocations, robertsonian translocations, and inversions) (5). However, its detection period is lengthy, it relies on cell culture, and it has low resolution, as it is unable to identify chromosomal abnormalities smaller than 10 Mb (3).

With the increasing popularity of chromosomal microarray analysis (CMA) and next-generation sequencing (NGS), the risks of CNVs have raised concerns among obstetricians and pregnant women (6, 7). Microdeletion/microduplication syndromes (MMS), including Williams syndrome and Prader–Willi syndrome, which are caused by pathogenic CNVs (pCNVs), can occur during any pregnancy and are not related to maternal age (8). MMS results in approximately 12% of unexplained intellectual disabilities, developmental delays, and various deformities (9). There are over 300 different MMS that have been clearly attributed to pCNVs, with a combined prevalence of nearly 1 in 600 (10). Research has shown that 6%–7% of fetuses with normal karyotyping but structural abnormalities detected by ultrasound have pCNVs or likely pCNVs (11). Furthermore, 1.0%–1.7% of fetuses with normal karyotyping and ultrasound results have pCNVs or likely pCNVs (12, 13).

Over the past 20 years, CMA has become a widely used method for diagnosing CNVs (14). Nevertheless, the large-scale application of CMA is limited due to its low throughput and high cost (11). Additionally, the limited coverage of probe arrays in CMA may result in some pCNVs not being detected (15). CNV-Seq, a method based on NGS, provides a novel way to identify CNVs. Its advantages include a wide detection range, high throughput, simple operation, a strong ability to detect chimerism, and low DNA sample requirements (16, 17). In 2019, Liu et al. published a Chinese expert consensus on the application of CNV-Seq to standardize its clinical use (18).

As CNV-Seq has gradually become a primary method for prenatal diagnosis, the changing target population for prenatal diagnosis warrants our attention. Invasive prenatal diagnostic procedures (IPDs) carry a certain risk of abortion; therefore, they are often recommended for pregnant women with clear prenatal diagnostic indications (19). Traditional prenatal diagnostic indications such as AMA and abnormal maternal serum screening (AMSS) generally indicate a high risk of chromosomal aneuploidy rather than CNV. In the context of CNV-Seq, the target population for prenatal diagnosis may change. Although some studies have shown that combining CNV-Seq with karyotyping can improve detection resolution while also detecting balanced chromosomal structural abnormalities such as inversions and translocations (11, 20), there is a lack of sufficient evidence regarding the value of the combined application of both techniques, including the effectiveness of detecting low-level mosaicism, the ability to detect CNVs effectively at chromosomal breakpoints for structural abnormalities, and cost considerations. More clinical data are needed to support the combined use of these two techniques.

In this study, the prenatal diagnosis results of 3,931 pregnant women (3,952 amniotic fluid samples) in the karyotype, Karyotype and CNV-Seq, and CNV-Seq groups were analyzed. This study clearly defined the detection efficiency of karyotyping and CNV-Seq for different types of chromosomal abnormalities, as well as their complementary roles, and evaluated the strengths and weaknesses of different prenatal diagnosis strategies.

## 2 Methods and materials

### 2.1 Subjects

**FIGURE 1:**
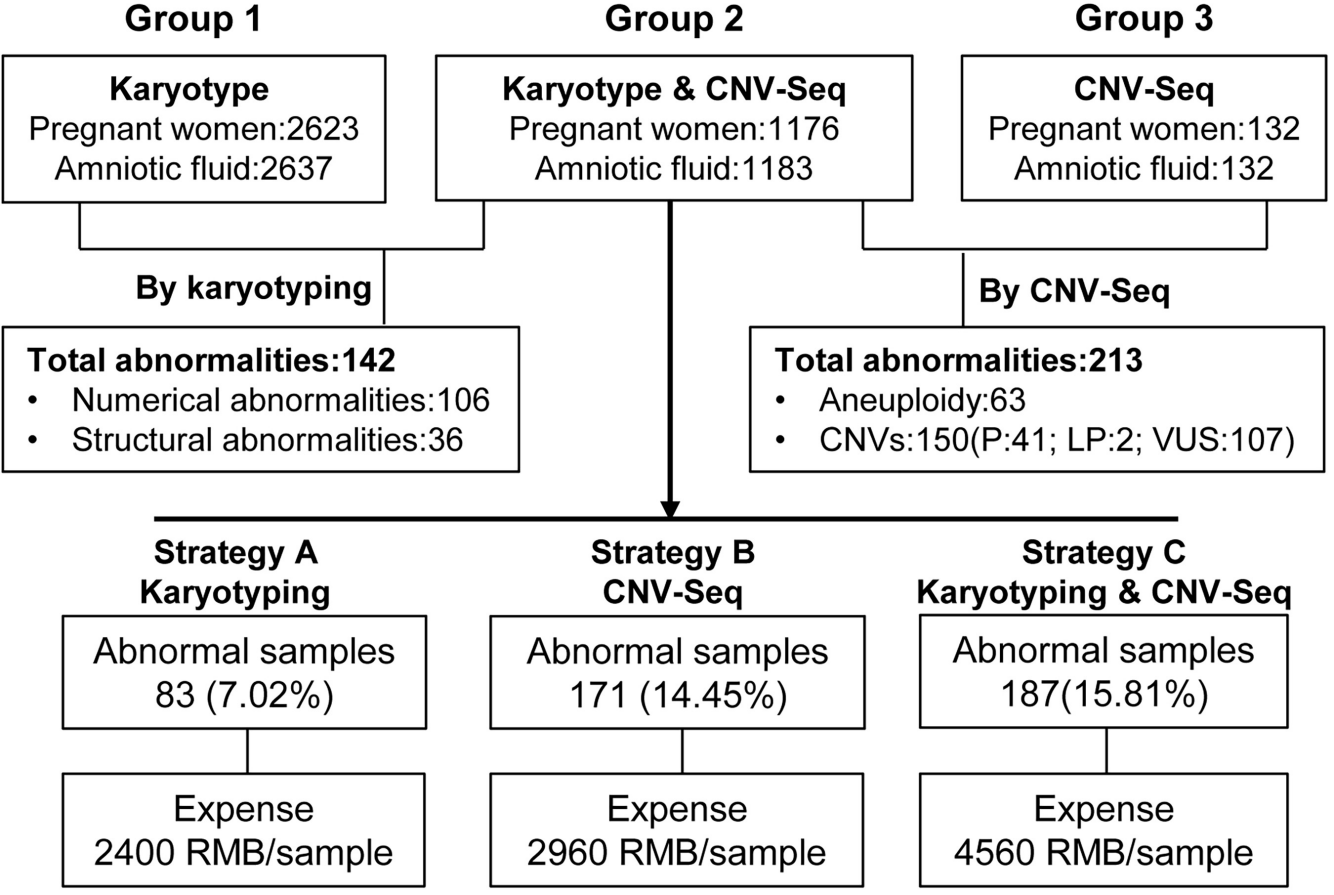
Flowchart of the three prenatal diagnosis groups and the three prenatal diagnosis strategies. CNV-Seq, copy number variant sequencing; CNVs, copy number variants; P, pathogenic; LP, likely pathogenic; VUS, variants of uncertain significance mutations.

This is a retrospective study conducted from November 2019 to October 2024, involving a total of 3,952 pregnant women who underwent invasive prenatal diagnostic procedures (amniocentesis) at the Central Hospital in Luohe, China. Among them, 21 pregnant women were excluded from the study due to missing clinical data or loss to follow-up, leaving 3,931 pregnant women included in the study. The age of the pregnant women in this study was 31 (21, 22) years; gestational age was 20 (19, 23) weeks. The 3,931 pregnant women (3,952 amniotic fluid samples) were divided into three groups on the basis of different genetic testing protocols: the karyotype group (undergoing only karyotyping), the Karyotype and CNV-Seq group (undergoing both karyotyping and CNV-Seq), and the CNV-Seq group (undergoing only CNV-Seq). The pregnant women included in this study and the results of prenatal diagnosis are summarized in Figure 1. Prior to amniocentesis, each pregnant woman received thorough genetic counseling, including information regarding the risks of amniocentesis and the advantages and limitations of various techniques for detecting chromosomal abnormalities in the fetus. All participants in this study provided informed consent and agreed to have their test results used for scientific research after their personal information was removed. This study adheres to strict privacy protection principles and was approved by the medical ethics committee of Luohe Central Hospital (number MEC-2019-026, approved on 20 June 2019).

All prenatal diagnostic indications for pregnant women were classified into 11 categories, including AMA, AMSS, twin pregnancy, ultrasonic anomalies (UA), either mother or father with chromosomal abnormality (EMFCA), non-invasive prenatal testing (NIPT), previous fetus/child with abnormalities (PFA), *in vitro* fertilization (IVF), teratogenic factors, voluntary diagnosis and family genetic history. The details for each prenatal diagnostic indication are provided in Table 1. Basic information and test results, including prenatal diagnostic indications, age, gestational age (GA), history of adverse pregnancies, and ultrasound examination results, were collected from the patients.

**TABLE 1:** Distribution of prenatal diagnostic indications in different groups.

Groups	Karyotype group		Karyotype and CNV-Seq group		CNV-Seq group		Total	
	*n*	%	*n*	%	*n*	%	*n*	%
Pregnant women	2,623	66.73	1,176	29.92	132	3.36	3,931	100.00
**Prenatal diagnostic indications**								
AMA	1,549	59.05	514	43.71	20	15.15	2,083	52.99
AMSS	999	38.09	343	29.17	17	12.88	1,359	34.57
Twin pregnancy	17	0.65	10	0.85	0	0.00	27	0.69
UA	33	1.26	155	13.18	88	66.67	276	7.02
EMFCA	5	0.19	22	1.87	0	0.00	27	0.69
NIPT	7	0.27	77	6.55	10	7.58	94	2.39
PFA	21	0.80	49	4.17	8	6.06	78	1.98
IVF	0	0.00	3	0.26	1	0.76	4	0.10
Teratogenic factors	2	0.08	16	1.36	0	0.00	18	0.46
Voluntary diagnosis	7	0.27	2	0.17	0	0.00	9	0.23
Family genetic history	1	0.04	4	0.34	1	0.76	6	0.15
**Details of 11 prenatal diagnostic indications**								
AMA	Advanced maternal age, 35 years or more							
AMSS	Abnormal maternal serum screening. High risk: trisomy 21 ≥ 1/270, trisomy 18 ≥ 1/350; Intermediate risk: 1/1,000 ≤ trisomy 21 ≤ 1/270, 1/1,000 ≤ trisomy 18 ≤ 1/350							
Twin pregnancy	Twin pregnancy or twin pregnancy became single pregnancy							
UA	Ultrasonic anomalies							
EMFCA	Either mother or father with chromosomal abnormality							
NIPT	High risk for chromosomal aneuploidy or copy number variations in non-invasive prenatal testing							
PFA	Previous fetus/child with abnormalities							
IVF	*In vitro* fertilization							
Teratogenic factors	Exposed to teratogenic factors or taking medication during pregnancy							
Voluntary diagnosis	Prenatal diagnosis without high risk factor							
Family genetic history	1. Family genetic history; 2. Pregnant women with intellectual disability							

Pregnant women with two indications were included in different groups for repeated statistical analysis. CNV-Seq, copy number variant sequencing.

### 2.2 Amniocentesis

Twenty milliliters of amniotic fluid was extracted under ultrasound guidance for further analysis. Currently, there are three main laboratory testing approaches for common chromosomal abnormalities in amniotic fluid samples: (1) karyotyping; (2) karyotyping and CNV-Seq; (3) CNV-Seq. According to expert consensus (18), it is recommended that all pregnant women undergoing invasive prenatal diagnosis should receive CNV-Seq testing, especially when there are fetal ultrasound abnormalities present (21). CNV-Seq can detect submicroscopic variations that traditional karyotyping cannot identify, thereby enhancing diagnostic accuracy (22). However, the final choice of the testing protocol still needs to consider the gestational age and the personal preferences of the pregnant woman. Pregnant women between 17 and 28 weeks of gestation can opt for either standalone fetal chromosomal karyotyping or a combined strategy of karyotyping and CNV-Seq based on their informed decision. For pregnant women beyond 28 weeks of gestation, due to time constraints on cell culture, rapid testing is usually conducted using CNV-Seq only (23).

For karyotyping, the amniotic fluid was cultured on the day of the puncture surgery, whereas the amniotic fluid was stored at 4 °C and genomic DNA extraction was performed within 48 h for CNV-Seq. Short tandem repeat (STR) testing was performed on all amniotic fluid samples for common chromosome aneuploidies (13/18/21/XY) for rapid diagnosis and exclusion of possible maternal cell contamination (MCC). In the case of MCC, the amniotic fluid was cultured for 10 days prior to CNV-Seq (24).

### 2.3 Amniotic fluid chromosome karyotyping

Following the centrifugation of approximately 15 mL of amniotic fluid and its inoculation into two culture bottles, the samples underwent culture, harvesting, and G-banding procedures (25). Chromosome images were obtained using a Zeiss automated chromosome scanner Imager Z2 (Carl Zeiss, Jena, Germany), and karyotyping was conducted using the provided chromosome analysis system MetaClient. At least 30 metaphase cells were counted for each sample, and a minimum of five karyotypes were analyzed. In the setting of chimerism, a count of 30–50 metaphase cells were conducted on the basis of the involved chromosomes. The karyotypes were described in accordance with the International System for Human Cytogenomic Nomenclature (ISCN) 2020 (26).

### 2.4 Copy number variation sequencing

One part of the CNV-Seq conducted in this study was completed by detection companies and the other part was independently carried out at Luohe Central Hospital. At Luohe Central Hospital, genomic DNA was extracted from the amniotic fluid using the QIAamp DNA Mini Kit (Qiagen, Valencia, CA, USA). Then, the CNVs detection kit, NGS library construction, and DNA purification reagents (Berry Genomics, Beijing, China), were used for NGS library construction, purification, and quality control. The NGS and bioinformatics analyses were conducted using the NextSeq CN500 sequencer (Illumina, San Diego, CA, USA), which generated approximately 5 million raw sequencing reads. The genome DNA sequence length was 36 bp, with an average sequencing depth of 0.1×.

The human reference genome sequence version GRCh37 was selected, and sequencing data were analyzed using the CNV detection algorithm developed by Tattini et al. (27), which detected CNVs with a resolution of 100 kb. In accordance with the American College of Medical Genetics (ACMG) guidelines, the pathogenicity of CNVs was evaluated by consulting public databases such as Clingen, DECIPHER, DGV, OMIM, NCBI, and UCSC. The clinical significance of CNVs is classified into five levels: benign (B), likely benign (LB), variants of uncertain significance (VUS), likely pathogenic (LP), and pathogenic (P) (28, 29). If a deletion or duplication region contained key areas associated with known MMS, carried an OMIM pathogenic gene, or had clinical significance in familial inheritance of phenotypic abnormalities reported in multiple studies or databases, it was defined as a P or LP CNV (30). B/LB CNVs are considered normal genetic polymorphisms, unrelated to clinical phenotypes. When a CNV lacks evidence to definitively determine if it is P/LP or B/LB, it is classified as a VUS. When a fetus is found to have a VUS CNV, genetic counselors will recommend that both the father and mother undergo CNV-Seq testing to determine if the mutation is inherited from the parents or a *de novo* mutation.

### 2.5 Statistics and reproducibility

All data analyses were conducted using IBM SPSS 25.0 software (IBM Corporation, Armonk, NY, USA). Categorical variables are described as numbers (%), and continuous variables are described as medians (lower quartile-upper quartile). When two or more sets of rates were compared, a chi-square test was used to determine if there was a statistically significant difference, with a significance level of *p* < 0.05. Pregnant women with two prenatal diagnostic indications were categorized into different groups for repeated statistical analysis. If two CNVs were found in the same sample, they were counted separately for analysis. In this study, the CNVs included for analysis included P, LP, and VUS variants, whereas B and LB variants were not included in the analysis.

## 3 Results

### 3.1 General characteristics and detection rates of chromosomal abnormalities in different groups

**FIGURE 2:**
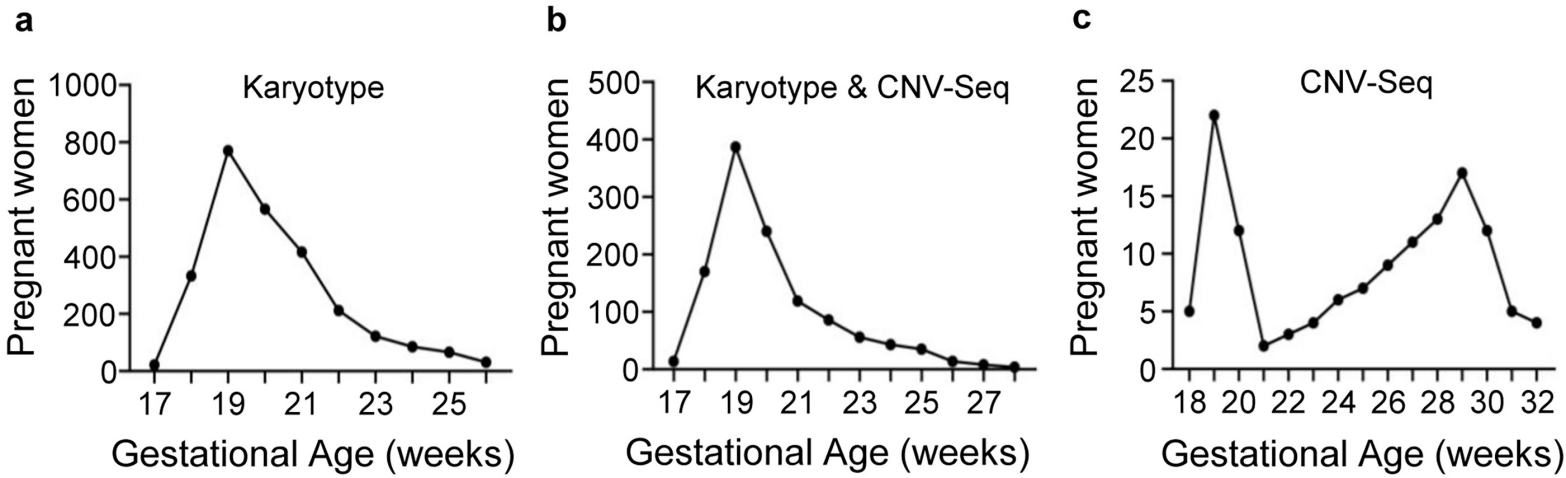
Gestational week distribution of pregnant women in the three groups who underwent prenatal diagnosis. **(a)** Karyotype group; **(b)** Karyotype and CNV-Seq group; **(c)** CNV-Seq group.

The study included 3,931 pregnant women. The patients’ general characteristics are described in Table 2. The numbers of pregnant women in the karyotype, Karyotype and CNV-Seq, and CNV-Seq groups were 2,623, 1,176, and 132, respectively. In the karyotype and Karyotype and CNV-Seq groups, 30.96% (1,176/3,799) of pregnant women chose to undergo CNV-Seq combined with karyotyping, indicating a certain level of acceptance of CNV-Seq among pregnant women. The three groups included 2,637, 1,183, and 132 samples of amniotic fluid, respectively. None of the pregnant women experienced any miscarriages related to amniocentesis. Among a total of 3,952 samples, 56 were brown in color and 85 had visible blood. The median age of the pregnant women in the three groups was 32.5, 32, and 30 years, respectively, while the median GA at the time of amniocentesis was 20, 20, and 26 weeks, respectively. The distribution of GA among pregnant women who underwent amniocentesis in the three groups is shown in Figure 2. Pregnant women in the karyotype and the Karyotype and CNV-Seq groups underwent amniocentesis mostly between 18–20 weeks of GA, with 19 weeks being the most common. However, pregnant women in the CNV-Seq group underwent amniocentesis at two main time points, 19 and 29 weeks of gestation.

**TABLE 2:** General characteristics and detection rates of chromosomal abnormalities in different groups.

Groups		Karyotype group	Karyotype and CNV-Seq group	CNV-Seq group	Total
Pregnant women		2,623	1,176	132	3,931
Amniotic fluid		2,637	1,183	132	3,952
Twins-monochorionic monoamniotic		3	3	0	6
Twins-dichorionic diamniotic		14	7	0	21
Maternal age (years) median (lower quartile-upper quartile)		32.5 (31, 34.5)	32 (29.5, 36)	30 (28, 33)	31 (28, 35)
Gestational age (weeks) median (lower quartile-upper quartile)		20 (19, 21)	20 (19, 21)	26 (20, 29)	20 (19, 21)
Abnormal karyotype	*n*	59	83	NA	142
	%	2.24 (59/2,637)	7.02 (83/1,183)	NA	3.72 (142/3,820)
	*p*	0.00		NA	NA
Chromosomal polymorphism	*n*	132	61	NA	193
	%	5.01 (132/2,637)	5.16 (61/1,183)	NA	5.05 (193/3,820)
	*p*	0.84		NA	NA
Aneuploidy in CNV-Seq	*n*	NA	60	3	63
	%	NA	5.07 (60/1,183)	2.27 (3/132)	4.79 (63/1,315)
	*P*	NA	0.15		NA
CNVs in CNV-Seq	*n*	NA	129	21	150
	%	NA	10.90 (129/1,183)	15.91 (21/132)	11.41 (150/1,315)
	*p*	NA	0.09		NA
Total abnormalities in CNV-Seq	*n*	NA	189	24	213
	%	NA	15.98 (189/1,183)	18.18 (24/132)	16.20 (213/1,315)
	*p*	NA	0.51		NA

CNV-Seq, copy number variant sequencing; CNVs, copy number variants; NA, not applicable.

The Karyotype and CNV-Seq group had a significantly greater DR of abnormal karyotypes than did the karyotype group (7.02% vs. 2.24%, *p* = 0.00). However, there was no statistically significant difference between the two groups in terms of chromosomal polymorphism DRs (5.01% vs. 5.16%, *p* = 0.84). Among pregnant women who underwent CNV-Seq, the DR of aneuploidy in the Karyotype and CNV-Seq group was greater than that in the CNV-Seq group, though the difference was not statistically significant (5.07% vs. 2.27%, *p* = 0.15). The CNVs detected in the CNV-Seq group exhibited a higher DR compared to the Karyotype and CNV-Seq group, though the difference was not statistically significant (15.91% vs. 10.99%, *p* = 0.09).

### 3.2 Distribution of prenatal diagnostic indications and detection rates of chromosomal abnormalities for different indications

The prenatal diagnostic indications for the three groups are listed in Table 1. Among all of the pregnant women, the highest percentage of indications was AMA (2,083, 52.99%), followed by AMSS (1,359, 34.57%), and UA (276, 7.02%), indicating that pregnant women with AMA are currently the primary target population for prenatal diagnosis. The indications of the three groups of pregnant women differed. In the karyotype group, AMA and AMSS accounted for 97.14% of all indications, whereas UA represented only 1.26%. In the karyotype and CNV-Seq group, the proportion of AMA and AMSS was 72.88%, whereas that of UA reached 13.18%. The highest proportion of indications in the CNV-Seq group was UA (88, 66.67%). The DRs of chromosomal abnormalities with different indications are shown in Table 3. Key prenatal diagnostic indicators with critical predictive roles for aneuploidy are EMFCA, NIPT and AMA, whereas for CNVs, they are UA, NIPT, and PFA.

**TABLE 3:** Detection rates of chromosomal abnormalities for different prenatal diagnostic indications.

Indications	Abnormal karyotype		Aneuploidy in CNV-Seq		CNVs in CNV-Seq	
	*n*	DR (%)	*n*	DR (%)	*n*	DR (%)
AMA	71	3.44	29	5.43	33	6.18
AMSS	44	3.28	14	3.89	35	9.72
Twin pregnancy	0	0.00	0	0.00	0	0.00
UA	5	2.66	4	1.65	42	17.28
EMFCA	10	37.04	2	9.09	2	9.09
NIPT	13	15.48	14	16.09	13	14.94
PFA	0	0.00	0	0.00	6	10.53
IVF	0	0.00	0	0.00	0	0.00
Teratogenic factors	1	5.56	0	0.00	0	0.00
Voluntary diagnosis	0	0.00	0	0.00	0	0.00
Family genetic history	0	0.00	0	0.00	2	40.00

Pregnant women with two indications were included in different groups for repeated statistical analysis. CNV-Seq, copy number variant sequencing; AMA, advanced maternal age; AMSS, abnormal maternal serum screening; UA, ultrasonic anomalies; EMFCA, either mother or father with chromosomal abnormality; NIPT, non-invasive prenatal testing; PFA, previous fetus/child with abnormalities; IVF, *in vitro* fertilization.

### 3.3 Distribution of abnormal chromosome karyotypes

In this study, a total of 3,820 samples were subjected to karyotyping, resulting in a 100% success rate for culture. Among them, 142 abnormal karyotypes were detected (3.72%), including 106 numerical abnormalities (2.77%) and 36 structural abnormalities (0.94%), as detailed in Table 4.

**TABLE 4:** Distribution of abnormal chromosome karyotypes in this study.

Abnormal chromosome karyotypes	Cases	
	*n*	%
Numerical abnormalities	106	2.77
Common trisomies	73	1.91
Trisomy 21/mosaic trisomy 21	62	1.62
Trisomy 18	10	0.26
Trisomy 13	1	0.03
Sex chromosome aneuploidies	27	0.71
47,XXX	4	0.10
47,XYY	8	0.21
47,XXY	9	0.24
Mosaic sex chromosome aneuploidies	6	0.16
Rare autosomal aneuploidies	4	0.10
Small supernumerary marker chromosome	1	0.03
Triploidy	1	0.03
Structural abnormalities	36	0.94
Inversion	4	0.10
Translocation	20	0.52
Deletion	2	0.05
Derivative	10	0.26
Total abnormalities	142	3.72

A total of 2,637 amniotic fluid samples (2,623 pregnant women) from the karyotype group and 1,183 amniotic fluid samples (1,176 pregnant women) from the Karyotype and CNV-Seq group were included.

In terms of numerical abnormalities, there were a total of 73 common trisomies (T21/T18/T13; trisomy 21/trisomy 18/trisomy 13), accounting for 1.91% of all cases. T21 (including mosaic T21), T18, and T13 accounted for 62 cases (1.62%), 10 cases (0.26%), and 1 case (0.03%), respectively. T21 was the most common fetal chromosomal abnormality. A total of 27 cases of sex chromosome aneuploidies (SCAs) were identified, including 4 cases (0.10%) of 47,XXX, 8 cases (0.21%) of 47,XYY, and 9 cases (0.24%) of 47,XXY. There were also 6 cases (0.16%) of mosaic SCAs, including X/XX, X/XY, XX/XY, XXX/XX, and XXY/XY. This study also identified 4 cases of mosaic rare autosomal aneuploidies (RAAs) involving chromosomes 10, 20, 15, and 12. Additionally, the study identified one case of a small supernumerary marker chromosome (sSMC) (47,XN,+mar) and one case of triploidy (69,XXY).

In this study, a total of 36 chromosomal structural abnormalities were identified, including 20 cases of translocation (0.52%), 10 cases of derivative (0.26%), 4 cases of inversion (0.10%), and 2 cases of deletion (0.05%).

### 3.4 Distribution of abnormal CNV-Seq results

Among the 1,315 samples that underwent CNV-Seq in this study, 213 cases (16.20%) were found to have chromosomal abnormalities, including 63 cases (4.79%) with aneuploidy and 150 cases (11.41%) with CNVs, as shown in Table 5.

**TABLE 5:** Distribution of abnormal CNV-Seq results in this study.

Abnormal CNV-Seq results	Cases	
	*n*	%
Aneuploidy	63	4.79
Common trisomies	45	3.42
Trisomy 21 (mosaic trisomy 21 included)	39	2.97
Trisomy 18	5	0.38
Trisomy 13	1	0.08
Sex chromosome aneuploidies	16	1.22
XXX	1	0.08
XYY	4	0.30
XXY	7	0.53
Mosaic sex chromosome aneuploidies (X/XX; XXX/XX; XXY/XY)	4	0.30
Rare autosomal aneuploidies (47,XN,+12[10%]/46,XN[90%]; 47,XN,+20[20%]/46,XN[80%])	2	0.15
CNVs	150	11.41
Deletions	63	4.79
Pathogenic	35	2.66
Likely pathogenic	1	0.08
Variants of uncertain significance	27	2.05
Duplications	87	6.62
Pathogenic	6	0.46
Likely pathogenic	1	0.08
Variants of uncertain significance	80	6.08
Total abnormalities	213	16.20

A total of 132 amniotic fluid samples (132 pregnant women) from the CNV-Seq group and 1,183 amniotic fluid samples (1,176 pregnant women) from the Karyotype and CNV-Seq group were included. CNV-Seq, copy number variant sequencing; CNVs, copy number variants.

In aneuploid cases, there were 45 common trisomies (3.42%), including 39 T21 (including mosaic T21) (2.97%), 5 T18 (0.38%), and 1 T13 (0.08%). Among 16 cases of SCAs, 1 case (0.08%) was 47,XXX, 4 cases (0.30%) were 47,XYY, 7 cases (0.55%) were 47,XXY, and 4 cases (0.30%) were mosaic SCAs. Two cases (0.15%) of mosaic RAAs were identified: 47,XN,+12 [10%]/46,XN [90%] and 47,XN,+20 [20%]/46,XN [80%].

CNV-Seq identified 150 cases (11.41%) of CNVs, including 63 cases (4.79%) of deletions and 87 cases (6.62%) of duplications. Among the 63 cases of deletions, there were 35 cases (2.66%) of deletion-P, 1 case (0.08%) of deletion-LP, and 27 cases (2.05%) of deletion-VUS. Among the 87 cases of duplications, 6 cases (0.46%) were duplication-P, 1 case (0.08%) was duplication-LP, and 80 cases (6.08%) were duplication-VUS. According to the CNV-Seq results, the most common aneuploidy was T21, whereas the most frequent CNV was duplication-VUS.

### 3.5 Comparison of the detection efficiency of karyotyping and CNV-Seq in the Karyotype and CNV-Seq group

Among the 1,183 amniotic fluid samples in the Karyotype and CNV-Seq group, we conducted a detailed comparison of the detection efficiency of the two techniques for different types of chromosomal abnormalities, as shown in Table 6.

**TABLE 6:** Comparison of the detection efficiency of karyotyping and CNV-Seq in the Karyotype and CNV-Seq group.

Karyotypes	CNV-Seq results	Cases	
		*n*	%
46,XN (polymorphisms included)	46,XN	994	84.02
46,XN (polymorphisms included)	Abnormal CNV-Seq results	102	8.62
	Duplications-p	6	0.51
	Duplications-VUS	62	5.24
	Deletions-P	26	2.20
	Deletions-LP	1	0.08
	Deletions-VUS	20	1.69
Balanced abnormalities	46,XN	12	1.01
46,XN,inv(9)(q22.3q32); 46,XN,inv(4)(p16q21); 46,XN,t(5;11)(q32;q21); 46,XN,t(3;5)(p23;q11.2); 46,XN,t(3;6)(p25;p22.2); 46,XN,t(9;16)(q22;q22); 46,XN,t(4;6)(q21;p23); 46,XN,t(6;18)(q34;q22); 46,XN,t(3;20)(q26.2;q11.2); 46,XN,t(5;10)(p13;q21); 45,XN,der(13;14)(q10;q10); 45,XN,der(14;21)(q10;q10)			NA
Balanced abnormalities	Abnormal CNV-Seq results (P/LP/VUS)	4	0.34
46,XN,t(3:14)(p21;q22)	del6q22.31 (120.72 Mb–121.18 Mb)	1	NA
46,XN,t(10;19)(p10;q10)	dup6q22.1 (117.40 Mb–117.64 Mb)	1	NA
46,XN,t(4;14)(q31.1;q32)	del22q11.22 (22.32 Mb–22.58 Mb)	1	NA
45,XN,der(6)t(6;13)(q27;q10),−13	del6q27 (169.24 Mb–170.92 Mb)	1	NA
Unbalanced abnormalities	46,XN	4	0.34
47,XN,+mar		1	NA
47,XN,+10[5]/46,XN[37]		1	NA
45,XN,−15[2]/46,XN[64]		1	NA
45,X[2]/46,XY[57]		1	NA
Unbalanced abnormalities	Abnormal CNV-Seq results (P/LP/VUS)	63	5.33
47,XN,+21	47,XN,+21	30	NA
Mosaic T21	1 of T21 and 3 of mosaic T21	4	NA
47,XN,+18	47,XN,+18	5	NA
47,XN,+13	47,XN,+13	1	NA
47,XYY	47,XYY	4	NA
47,XXY	47,XXY	6	NA
47,XXX	47,XXX	1	NA
Mosaic SCAs (X/XX, XX/XXX, XY/XXY)	Mosaic SCAs (X/XX, XX/XXX, XY/XXY)	3	NA
47,XN,+12[2]/46,XN[52]	47,XN,+12[10%]/46,XN[90%]	1	NA
47,XN,+20[44]/46,XN,[54]	47,XN,+20[20%]/46,XN[80%]	1	NA
46,XN,der(21;21)(q10;q10),+21	47,XN,+21	1	NA
46,XN,del(5)(15.2)	del5p15.33p15.2 (0.02 Mb–13.36 Mb)	1	NA
47,XN,+21	47,XN,+21	1	NA
	dup(X)(p11.21p11.1) (56.84 Mb–58.56 Mb)		NA
47,XN,+21	47,XN,+21	1	NA
	del 5q23.1q23.1 (120.38 Mb–120.98 Mb)		NA
47,XXY	47,XXY	1	NA
	dup(X)(p22.31p22.31) (6.42 Mb–8.14 Mb)		NA
46,N,del(X)(q26)	delXq27.1q28 (138.56 Mb–154.92 Mb)	1	NA
	dup6q14.1 (80.12 Mb–80.34 Mb)		NA
46,XN,der(4)t(3;4)(p21;p16)	dup3p26.3p21.31 (0.06 Mb–46.56 Mb)	1	NA
	del4p16.3p16.3 (0.08 Mb–2.24 Mb)		NA
Unidentified karyotypes	CNV-Seq results	4	0.34
46,XN,?9	dup(9)(p21.2p21.1) (26.70 Mb–32.22 Mb)	1	NA
46,XN,?8	del8p21.2p11.23 (24.18 Mb–38.20 Mb)	1	NA
46,XN,?16	46,XN	1	NA
46,XN,?16	46,XN	1	NA

CNV-Seq, copy number variant sequencing; P, pathogenic; LP, likely pathogenic; VUS, variants of uncertain significance mutations; NA, not applicable.

In 994 samples (84.02%), no abnormalities were detected via karyotyping or CNV-Seq. In 102 samples, the karyotyping results revealed 46,XN or chromosomal polymorphisms, whereas the CNV-Seq results revealed P/LP/VUS CNVs (CNV variant loci unrelated to chromosomal polymorphisms). The largest size of CNVs in these 102 samples was 9.56 Mb, which exceeded the detection range of karyotyping. Among 12 samples (1.01%), karyotyping revealed balanced abnormalities (including inversions, balanced translocations, and derivative chromosomes), whereas CNV-Seq results did not reveal any abnormalities.

Among four samples (0.34%), karyotyping revealed balanced abnormalities (balanced translocations and derivative chromosomes), whereas CNV-Seq revealed CNVs with sizes smaller than 1 Mb. Notably, one patient presented with a derivative chromosome, with a karyotyping result of 45,XN,der(6)t(6;13)(q27;q10),−13, whereas the CNV-Seq result indicated a deletion at 6q27 (169.24 Mb–170.92 Mb). Although the CNV was smaller than the resolution of karyotyping, the fact that the location of the CNV matches the chromosome breakpoints identified by karyotyping indicates that the CNV occurred at the breakpoints of the chromosomes.

In four samples (0.34%), karyotyping revealed unbalanced abnormalities, whereas the CNV-Seq results revealed no abnormalities. This included one case of sSMC (47,XN,+mar). It is speculated that the extra +mar chromosome is composed of heterochromatin that cannot be effectively detected by CNV-Seq. This was later confirmed by C-banding analysis. Karyotyping revealed mosaic RAAs (47,XN,+10[5]/46,XN[37] and 45,XN,−15[2]/46,XN[64]) in two samples and mosaic SCAs (45,X[2]/46,XY[57]) in one sample. However, the CNV-Seq results did not reveal any abnormalities, indicating that CNV-Seq failed to detect low-level mosaicism in these three cases.

Among the 1,183 samples, 63 (5.33%) were found to have abnormal results via both karyotyping and CNV-Seq, including 37 cases of T21 (including translocation T21 and mosaic T21), 5 cases of T18, 1 case of T13, 15 cases of SCAs (including mosaic SCAs), and 2 cases of mosaic RAAs. The karyotyping results of three amniotic fluid samples revealed deletions or derivative chromosomes [46,XN,del(X)(q26); 46,XN,del(5)(15.2); 46,XN,der(4)t(3;4)(p21;p16)], and the CNV-Seq results confirmed the corresponding abnormal karyotypes.

**FIGURE 3:**
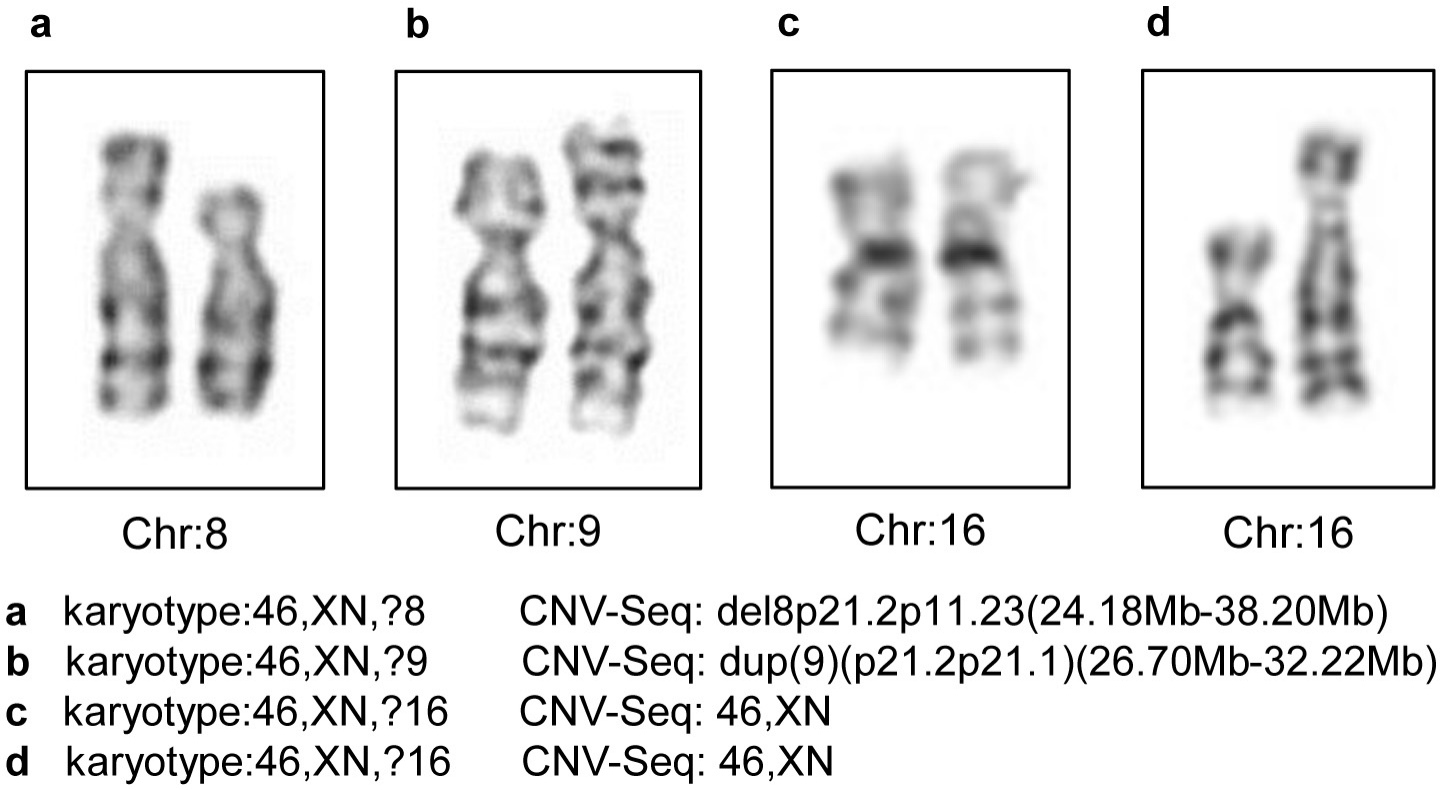
The four unidentified karyotypes and the CNV-Seq results. Chr, chromosome; CNV-Seq, copy number variant sequencing.

Through CNV-Seq, a total of four cases with unidentified karyotypes were successfully validated, including one case with a karyotype of 46,XN,?9, though the CNV-Seq results revealed a duplication of 9p21.2p21.1 (26.70 Mb–32.22 Mb). One sample had a karyotype of 46,XN,?8, while the CNV-Seq result was del8p21.2p11.23 (24.18 Mb–38.20 Mb). Additionally, there were two samples with a karyotype of 46,XN,?16 but CNV-Seq results of 46,XN. The karyotype images of the four unidentified karyotypes are shown in Figure 3.

### 3.6 Comparison of the detection efficiency of karyotyping and CNV-Seq for chimerism

In this study, 13 sample of amniotic fluid in the Karyotype and CNV-Seq group were found to have chimerism through karyotyping or CNV-Seq, including 4 cases of T21, 4 cases of SCAs, 4 cases of RAAs, and 1 case of mosaic deletion, as shown in Table 7. CNV-Seq revealed a mosaic deletion of 30.84 Mb {del8p23.3p12 (0.16 Mb–31.00 Mb); 46,XN, [90%]/46,XN, del8p23.3p12 [10%]}, which was not detected by karyotyping. Two cases of mosaicism (45,XN,−15[2]/46,XN[64] and 45,X [2]/46,XY [57]) were identified through karyotyping but were not detected by CNV-Seq. In this study, the lowest level of mosaicism detected by karyotyping was 3.03%, whereas that detected by CNV-Seq was 10%.

**TABLE 7:** Comparison of the detection efficiency of karyotyping and CNV-Seq for chimerism.

Karyotyping		CNV-Seq	
Result	Mosaicism level (%)	Result	Mosaicism level (%)
45,X[7]/46,XX[45]	13.46	del(X)(p22.33q28) (1–155.27 Mb); 45,X[10%]/46,XX[90%]	10.00
47,XN,+21[2]/46,XN[44]	4.35	dup21q11.2q22.3 (14.60 Mb–48.10 Mb); 47,XN,+21[15%]/46,XN[85%]	15.00
47,XXX[34]/46,XX[33]	50.75	dupXp22.33q28 (0.06 Mb–155.26 Mb); 46,XX[79%]/47,XXX[21%]	21.00
47,XN,+21[18]/46,XN[55]	24.66	dup21p11.2q22.3 (9.41 Mb–48.11 Mb); 47,XN,+21[22%]/46,XN[78%]	22.00
47,XN,+20[44]/46,XN,[54]	44.90	dup(20)(p13q13.33) (1–63.03 Mb); 47,XN,+20[20%]/46,XN[80%]	20.00
47,XN,+21[2]/46,XN[36]	5.26	dup(21)(q11.2q22.3) (14.30 Mb–48.13 Mb); 47,XN,+21(100%)	100.00
46,XN	NA	del8p23.3p12 (0.16 Mb–31.00 Mb); 46,XN,[90%]/46,XN,del8p23.3p12[10%]	10.00
47,XN,+21[5]/46,XN[48]	9.43	dup(21)(q11.2q22.3) (14.30 Mb–48.13 Mb); 47,XN,+21[30%]/46,XN[70%]	30.00
47,XN,+12[2]/46,XN[52]	3.70	dup(12)(p13.33q24.33) (1–133.85 Mb); 47,XN,+12[10%]/46,XN[90%]	10.00
47,XN,+10[5]/46,XN[37]	11.90	46,XN	0.00
47,XXY[13]/46,XY[37]	26.00	dup Xp22.33q28 (1–155.27 Mb); 47,XXY[50%]/46,XN[50%]	50.00
45,XN,–15[2]/46,XN[64]	3.03	46,XN	NA
45,X[2]/46,XY[57]	3.39	46,XN	NA

CNV-Seq, copy number variant sequencing; NA, not applicable.

### 3.7 Clinical value and economic cost of different prenatal diagnosis strategies

The clinical utility and economic costs of three different prenatal diagnosis strategies (only conducting karyotyping, only conducting CNV-Seq, and combining karyotyping and CNV-Seq) were evaluated, as depicted in Figure 1 and Table 8. The three prenatal diagnosis strategies had DRs for chromosomal abnormalities of 7.02%, 14.45%, and 15.81%, respectively. There was a statistically significant difference in the DRs of chromosomal abnormalities among the three prenatal diagnosis strategies (*p* = 0.00). The costs of each prenatal diagnosis strategy per sample were 2400 RMB, 2960 RMB, and 4560 RMB, respectively, including fees for amniocentesis. Compared with only conducting karyotyping, the cost of combining karyotyping and CNV-Seq increased by 90.00%, though the DR of chromosomal abnormalities increased by 125.21%. Therefore, the combination of karyotyping and CNV-Seq is considered a cost-effective prenatal diagnostic strategy.

**TABLE 8:** Clinical value and economic cost of different prenatal diagnosis strategies.

Strategies	Karyotyping			CNV-Seq			Karyotyping and CNV-Seq
	Numerical	Structural	Total	Aneuploidy	CNVs	Total	
Abnormal samples	63	20	83	62^a^	109^b^	171	187^a+b^
DR (%)	5.33	1.69	7.02	5.24	9.21	14.45	15.81
Expense	2400 RMB/sample			2960 RMB/sample			4560 RMB/sample

^a^5 cases of amniotic fluid contained both aneuploidy and CNVs and were classified as aneuploidy.

^b^13 cases of amniotic fluid contained 2 CNVs. CNV-Seq, copy number variant sequencing; CNVs, copy number variants; DR, detection rate.

## 4 Discussion

Similar to T21 and other aneuploidies, CNVs are important causes of birth defects. In theory, combining CNV-Seq with karyotyping can achieve comprehensive detection of aneuploidies, structural abnormalities, and CNVs. In this study, we conducted a statistical analysis of prenatal diagnostic indications in three diagnostic strategy groups to examine the changes in the target population for prenatal diagnosis in the setting of CNV-Seq. We compared the detection efficiency of karyotyping and CNV-Seq for various types of chromosomal abnormalities, assessing the effectiveness and costs of three prenatal diagnosis strategies. This study supports the recommendation for the broader use of CNV-Seq and the combined use of karyotyping and CNV-Seq.

As the awareness of eugenics and prenatal care is increasing, more pregnant women are choosing to undergo prenatal diagnosis. The indications for prenatal diagnosis are becoming more diverse, such as contracting COVID-19 during pregnancy or being exposed to teratogenic factors. In this study, we carefully classified the prenatal diagnostic indications and reported that AMA is the most common indication, whereas UA is the primary indication for pregnant women who only undergo CNV-Seq. The proportion of pregnant women with AMA was as high as 52.99% in this study, which is higher than the previously reported rate of 27.013%–39.1% (31–33). The main reason for the higher proportion of AMA pregnant women in this study compared to previous research is twofold. Firstly, in China, the proportion of AMA pregnant women is continuously increasing. For example, in the Luohe region of China where this study was conducted, the proportion of AMA pregnant women among all pregnant women increased from 11.30% in 2019 to 16.03% in 2023, which is later than in previous studies. Secondly, the local government in this region covers the prenatal diagnostic costs for some pregnant women, such as AMA or AMSS pregnant women, but not all pregnant women. Therefore, AMA pregnant women in this region are more willing to undergo prenatal diagnosis rather than NIPT compared to other regions. A comparison of the DRs of different indications for chromosomal abnormalities revealed that EMFCA, NIPT and AMA are predictive indicators for chromosomal aneuploidy, whereas UA is a predictive indicator for CNVs. Traditional prenatal diagnostic indications focus primarily on aneuploidy, whereas in the context of CNV-Seq, more attention should be given to pregnant women with fetal ultrasound abnormalities. In this study, the distribution of GA among pregnant women in the CNV-Seq group had two distinct peaks. The second peak (28–32 weeks) mainly included pregnant women who underwent prenatal diagnosis due to fetal ultrasound abnormalities in the late stage of pregnancy. The proportions of pregnant women with UA in the karyotype, Karyotype and CNV-Seq, and CNV-Seq groups were 1.26, 13.18, and 66.67%, respectively, indicating that pregnant women with ultrasound abnormalities are more likely to undergo CNV-Seq, which is consistent with previous reports (11).

The DR of abnormal karyotypes was 3.72% in this study, which is similar to the previously reported range of 2.88%–3.46% (31, 33–35). As the gold standard for diagnosing fetal chromosomal abnormalities, karyotyping has relatively stable detection performance. The most common chromosomal abnormality observed was T21, which is consistent with previous literature reports (36). One of the most significant advantages of karyotyping over molecular detection techniques such as CNV-Seq is its ability to detect chromosomal rearrangements across the entire genome (11), a capability that cannot be achieved by any other method such as CMA or CNV-Seq. In this study, karyotyping revealed 36 cases (0.94%) of chromosomal structural abnormalities, including inversions, translocations, and derivative chromosomes. Notably, there was a significant difference in the DR of abnormal karyotypes between the karyotype and the Karyotype and CNV-Seq groups (2.24% vs. 7.02%). This difference is attributed to the varying proportions of indications between the two groups. In a study by Xiao involving 12,365 amniotic fluid samples, the overall DR of the fetal karyotyping was 3.46%. The fetal karyotype abnormality rates for pregnant women with AMA and AMSS were 2.79% and 2.23%, respectively, both of which are lower than the average level. In this study, the combined proportion of pregnant women with AMA and AMSS in the karyotype group was 97.14%, whereas that in the Karyotype and CNV-Seq group was 72.88%.

CNV-Seq is capable of detecting chromosomal abnormalities greater than 100 kb, as well as mosaicism, with a mosaicism level as low as 5% (37, 38). Several studies have evaluated the utility and accuracy of CNV-Seq; however, there is a significant difference in the reported DR of CNV-Seq for prenatal diagnoses (39, 40). This variation may be related to the prenatal diagnostic indications of the study population, detection platform used, or abnormal reporting standards of CNVs. In this study, the overall DR of CNVs in pregnant women was 16.20%.

A comparison of the prenatal diagnostic results of 1,183 amniotic fluid samples subjected to karyotyping and CNV-Seq revealed that the combined use of CNV-Seq with karyotyping offers advantages over karyotyping alone. A previous study that CNV-Seq detected 347 cases of chromosomal abnormalities (13.80%) in 2,514 amniotic fluid samples with normal karyotypes (20). Similarly, Lan identified 123 cases of chromosomal abnormalities (13.47%) in 913 amniotic fluid samples with normal karyotypes (41). Therefore, the combined use of CNV-Seq with karyotyping improves the DR of chromosomal abnormalities. In this study, out of 1,183 samples, 102 had normal or polymorphic results via karyotyping, though CNV-Seq revealed P/LP/VUS mutations. In addition, CNV-Seq can be used to validate the polymorphic results identified by karyotyping. In this study, among the 61 samples in the Karyotype and CNV-Seq group, karyotyping revealed chromosomal polymorphisms, whereas CNV-Seq did not detect any CNVs related to polymorphic sites, confirming the accuracy of karyotyping. CNV-Seq can also be used to confirm unidentified karyotypes. In this study, four cases of unidentified karyotypes were confirmed through CNV-Seq. The resolution of karyotyping is generally believed to be 5–10 Mb (3), and variations in size close to the resolution of the karyotype or poor chromosome preparation can lead to misdiagnoses. Validation of the pathogenicity of the sSMC can also be achieved by combining karyotyping and CNV-Seq. sSMCs, also known as marker chromosomes, refer to extra chromosomes with recognizable morphology that cannot be identified using traditional karyotyping (26). Previous studies have suggested that the DR of fetal sSMCs is 0.8%–1.5% (11). In this study, a case of sSMC was identified through karyotyping, with CNV-Seq results showing 46,XN, indicating that the additional +mar chromosome was deemed non-functional heterochromatin.

Although some studies have shown that combining CNV-Seq with Karyotype can improve the DR of amniotic fluid chromosomal abnormalities, there is little mention of the economic effects of CNV-Seq technology by scholars. In this study, when comparing three different diagnostic strategies, it was found that Karyotype and CNV-Seq increased costs by 106.67%, but improved the DR of chromosomal abnormalities by 125.21% compared to Karyotype alone, making it a cost-effective diagnostic strategy. It is worth noting that when conducting CNV detection, choosing CNV-Seq over CMA can reduce the hospital’s costs in equipment and laboratory construction. With the widespread adoption of NIPT, using just one high-throughput sequencer can perform both NIPT and CNV-Seq simultaneously.

The limitations of CNV-Seq should not be overlooked. CNV-Seq is unable to detect triploidy, as triploid fetuses are more likely to miscarry in the first trimester but can also miscarry in the second trimester (42). In this study, the karyotype group included a triploid fetus (CNV-Seq was not performed). Prior to CNV-Seq, the use of STR detection can effectively detect triploidy (7). Similarly, as in this study, CNV-Seq is unable to detect balanced translocations, inversions, and other balanced structural abnormalities or to differentiate between free trisomy and translocation trisomy (11). Carriers of balanced translocations are more likely to produce unbalanced gametes, which can have an impact on the fertility of the next generation (43). However, true balanced translocations and inversions do not result in changes in gene dosage and, therefore, do not lead to the occurrence of birth defects (44). Additionally, 7.9% of samples with balanced translocations detected via karyotyping may have CNVs at the breakpoints, making them potentially detectable by CNV-Seq (45). In this study, karyotyping revealed derivative chromosomes in two samples, though CNVs related to the breakpoints were identified via CNV-Seq [45,XN,der(6)t(6;13)(q27;q10),−13; 46,XN,der(4)t(3;4)(p21;p16)]. Another limitation of CNV-Seq is that chimerism consisting of 47,XXX and 45,X in equal proportions of 50% each will be mistakenly identified as 46,XX. Also, CNV-Seq cannot identify loss of heterozygosity (LOH), including uniparental disomy (UPD) (46). CNV-Seq detection also has limitations in identifying highly repetitive regions, including certain chromosomal polymorphisms and sSMC chromosomes in this study (47). Last, CNV-Seq has poor resolution for highly homologous sequences, such as the presence of homologous sequences between the X and Y chromosomes, resulting in a limited ability to detect sex chromosome mosaicism (48).

Mosaicism is present in 1%–2% of samples obtained through chorionic villus sampling, whereas 0.1%–0.5% of amniotic fluid samples exhibit mosaicism (49). The ability to detect chimerism varies depending on the technology and detection platform used. CMA is unable to accurately analyze chimerism below 30%, whereas CNV-Seq can detect lower levels of chimerism (50). CNV-Seq has the ability to detect chromosomal aneuploid chimerism as low as 5% under ideal conditions (38). Cohen suggested that CNV-Seq is much better than aCGH for detecting chimerism (40), whereas Shi’s study revealed that CNV-Seq can detect low-level chimerism of 10%–20% (17). Previous studies suggest that karyotyping can detect chimerism as low as 5% (49). In general, CMA and CNV-Seq are more reliable than karyotyping for the diagnosis of chimerism as they do not require the culture of amniotic fluid cells (49). On the basis of the results of this study, karyotyping and CNV-Seq each have their own advantages in diagnosing chimerism. A total of 13 cases of chimerism were identified in Karyotype and CNV-Seq group, with karyotyping missing one case and CNV-Seq missing two cases. CNV-Seq detected three cases of chimerism with a mosaicism level of 10%, whereas karyotyping identified five out of 12 cases with a mosaicism level below 10%. Importantly, in the 12 cases of chimerism identified through karyotyping, the number of metaphases counted ranged from 38 to −98, all of which exceeded the standard requirement of 20 metaphases for conventional counting. To accurately identify chimerism in karyotyping, a sufficient number of metaphases must be counted.

This study has several limitations. The choice of amniocentesis testing approaches depends not only on prenatal diagnostic indications but also on gestational age and personal preferences. The cost of karyotyping is covered by the local government, while CNV-Seq expenses are borne by the pregnant woman, potentially leading to biased results. The sample size was small, leading to potential data bias in some of the detection indicators. In addition, due to missing data, this study did not focus on the follow-up of pregnancy outcomes after prenatal diagnosis. As a retrospective single-center analysis, the potential for selection and information biases cannot be fully excluded. To minimize these risks, we applied consistent inclusion and exclusion criteria and relied on standardized electronic medical records and laboratory protocols. Moreover, while propensity score matching or multivariate modeling was not applied in this descriptive study, stratified analyses by prenatal diagnostic indication were performed to partially control for confounding. Future prospective, multicenter investigations using advanced statistical adjustments are warranted to confirm and extend our findings.

## 5 Conclusion

In the era of CNV-Seq, the target population for prenatal diagnosis is expanding. Karyotyping and CNV-Seq have unique advantages for detecting different types of chromosomal abnormalities. Karyotyping excels in detecting chromosomal structural abnormalities, whereas CNV-Seq is effective in detecting microdeletions and microduplications. Both methods have the ability to detect low-level mosaicism to some extent. Combining karyotyping and CNV-Seq can increase the DRs of chromosomal abnormalities, making it a cost-effective prenatal diagnostic strategy that is both efficient and economical.

## Data Availability

The raw data supporting the conclusions of this article will be made available by the authors, without undue reservation.
